# Evolutionary trends of the conserved neurocranium shape in angel sharks (Squatiniformes, Elasmobranchii)

**DOI:** 10.1038/s41598-020-69525-7

**Published:** 2020-07-28

**Authors:** Faviel A. López-Romero, Sebastian Stumpf, Cathrin Pfaff, Giuseppe Marramà, Zerina Johanson, Jürgen Kriwet

**Affiliations:** 1 Department of Palaeontology, University of Vienna, Althanstraße 14, Geocenter, 1090 Vienna, Austria; 2 Dipartimento di Scienze Della Terra, Università Degli Studi di Torino, Via Valperga Caluso 35, 10125 Torino, Italy; 3 Department of Earth Sciences, Natural History Museum, Cromwell Road, London, SW75BD UK

**Keywords:** Ichthyology, Evolution, Palaeontology

## Abstract

Elasmobranchii (i.e., sharks, skates, and rays) forms one of the most diverse groups of marine predators. With a fossil record extending back into the Devonian, several modifications in their body plan illustrate their body shape diversity through time. The angel sharks, whose fossil record dates back to the Late Jurassic, some 160 Ma, have a dorsoventrally flattened body, similar to skates and rays. Fossil skeletons of this group show that the overall morphology was well established earlier in its history. By examining the skull shape of well-preserved fossil material compared to extant angel sharks using geometric morphometric methods, within a phylogenetic framework, we were able to determine the conservative skull shape among angel sharks with a high degree of integration. The morphospace occupation of extant angel sharks is rather restricted, with extensive overlap. Most of the differences in skull shape are related to their geographic distribution patterns. We found higher levels of disparity in extinct forms, but lower ones in extant species. Since angel sharks display a highly specialized prey capture behaviour, we suggest that the morphological integration and biogeographic processes are the main drivers of their diversity, which might limit their capacity to display higher disparities since their origin.

## Introduction

**Figure 1:**
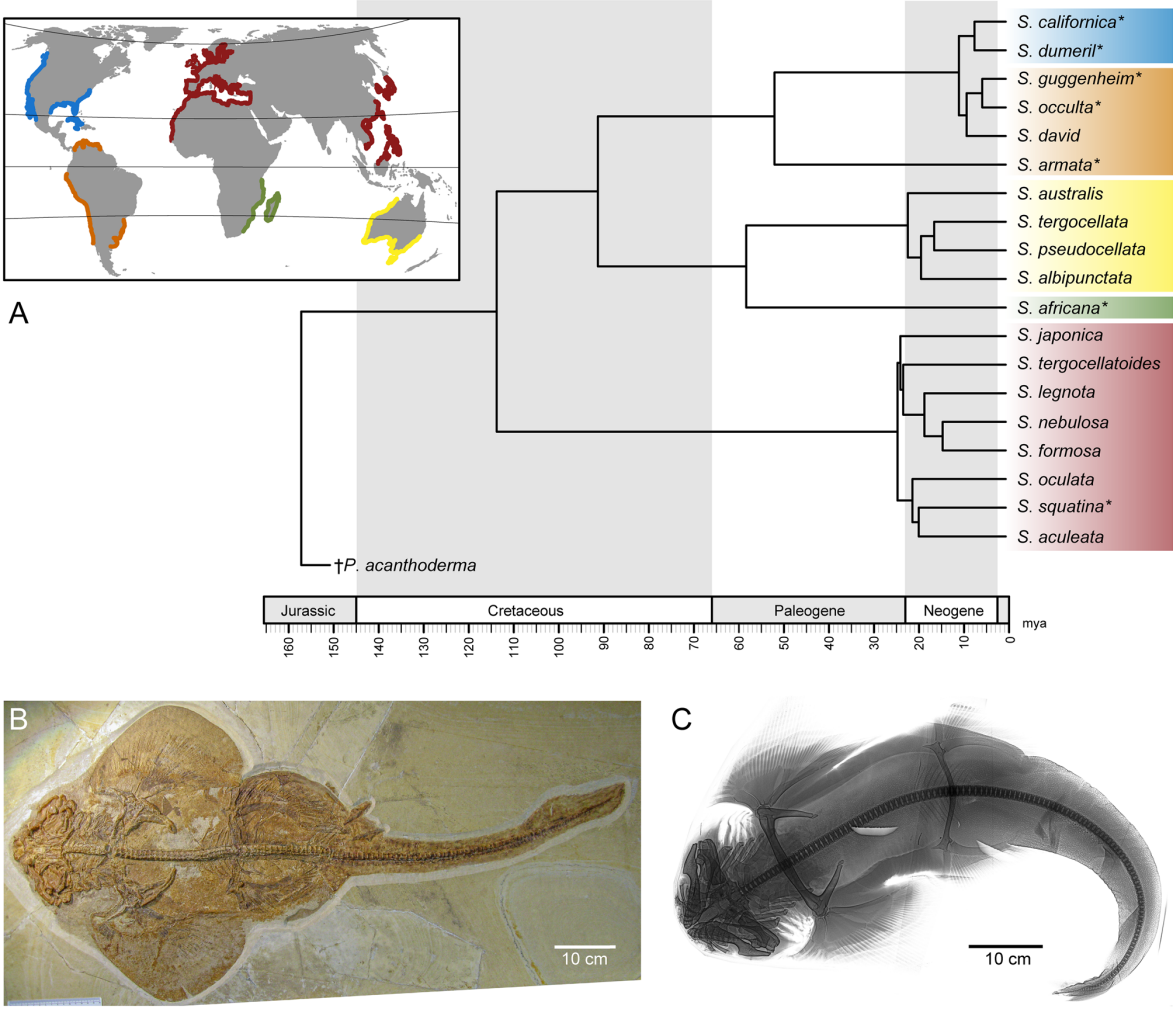
(**A**) Distribution and phylogeny of extant angel sharks worldwide, the colours correspond to the clades highlighted in the phylogeny. Phylogeny of the angel shark species, modified from Stelbrink et al.^17^ colour code indicates the respective clade, *represents the species used for the present study. (**B**) Fossil angel shark †*Pseudorhina acanthoderma*, adult specimen (SMNS 8621441), (**C**), Holotype X-Ray of *Squatina africana* (BMNH1906.11.19.21), modified to fit the figure and downloaded from https://data.nhm.ac.uk/object/15711224-7ceb-4829-95cb-4286fc14bcad/1591228800000.

Elasmobranchs (sharks, skates, and rays) represent a lineage of vertebrates with a fossil record extending over 400 million years^1,2^. Throughout their evolutionary history, adaptations of feeding mechanisms, behavioural specializations and ecological distributions are well reflected in their body plan^3,4^. Most modern sharks display rather conservative overall body shapes^5^, for most of the pelagic species, and distinguishable from the benthic species^6^, also some specific morphological traits like the neurocranium may vary strongly in shape, as seen in the hammerhead and sawsharks^7–10^. Another example of extreme morphology is displayed by angel sharks (Squatiniformes), which are characterized by highly dorsoventrally compressed bodies similar to batomorphs (i.e., skates and rays). This similarity caused controversies in their phylogenetic placement^11,12^, but their systematic position within sharks^13–16^ and intra-relationships^17^ are well resolved (Fig. 1). All the 22 living valid species of the squatiniforms are included in a single genus, *Squatina*^18–20^. Living angel sharks can be divided into clades that follow a biogeographical pattern explained both by vicariant and dispersion events during the last 20 million years, forming distinct Europe-North African, South African, Asian, Australian, North American and South American clades^17^. More recently, it has been suggested that the species from North and South America can be differentiated into eastern and western clades^21^.

The evolutionary history of angel sharks can be traced back into the Oxfordian (Late Jurassic; 160 Ma), with several specimens initially assigned to *Squatina,* but later transferred to the genus †*Pseudorhina* in the family †Pseudorhinidae, ranging from the Oxfordian to the Tithonian^22,23^. Extant members of the order Squatiniformes have a fossil record that dates back to the Early Cretaceous^23,24^. The body plan of Squatiniformes nevertheless, arose during the Late Jurassic, based on holomorphic fossil specimens, which remains consistent with only few changes to date (Fig. 1B,C). Another feature present in some of the holomorphic fossil species is the neurocranium, which usually is not preserved among cartilaginous fishes. The overall morphology of the dorso-ventral flattening, the extensive pectoral and pelvic fins, the number of dorsal fins, and the jaw position indicate a possible similarity in their ecology for both †*Pseudorhina* and *Squatina*, suggested to be near-coastal bottom-dwelling species^25^. Despite the remarkable features of the body plan of angel sharks, little attention has been paid to its possible origins as well as other aspects of their biology^26^. A previous work on the skeletal morphology of angel sharks has demonstrated that the neurocranial traits are useful to determine species^27^. Differences in the neurocranium are mainly expressed in the shape of the rostral region, which can be anteriorly extended. Because most of the other external morphological characters and even the dentition tends to be rather conservative, the taxonomic assignment of fossils to living species remains problematic^20,28–30^.

Morphological evolution is a process that comprises a series of constraints as well as ecological opportunities for organisms^31,32^. Additionally, the capacity of organisms to evolve can be determined by extrinsic and/or intrinsic factors throughout their lifetime and evolutionary history^33–35^. Possible phenotypic shapes that a particular structure can take are limited by various evolutionary constraints that may impose limits to their variability, like developmental processes, ecological opportunity and/or interactions with other organisms^36–38^. Adaptive radiations are major examples of how morphological evolution can take place in short periods of time, but also in restricted ranges of distribution^32,39,40^. However, morphological disparity does not always match speciation rates, probably as a result of distinct biogeographic patterns, such as climatic factors limiting distribution ranges^41^. A common trend observed in several studies indicates that some clades initially displayed a larger morphological disparity, which otherwise became much reduced through their evolutionary history^42–45^.

**Figure 2:**
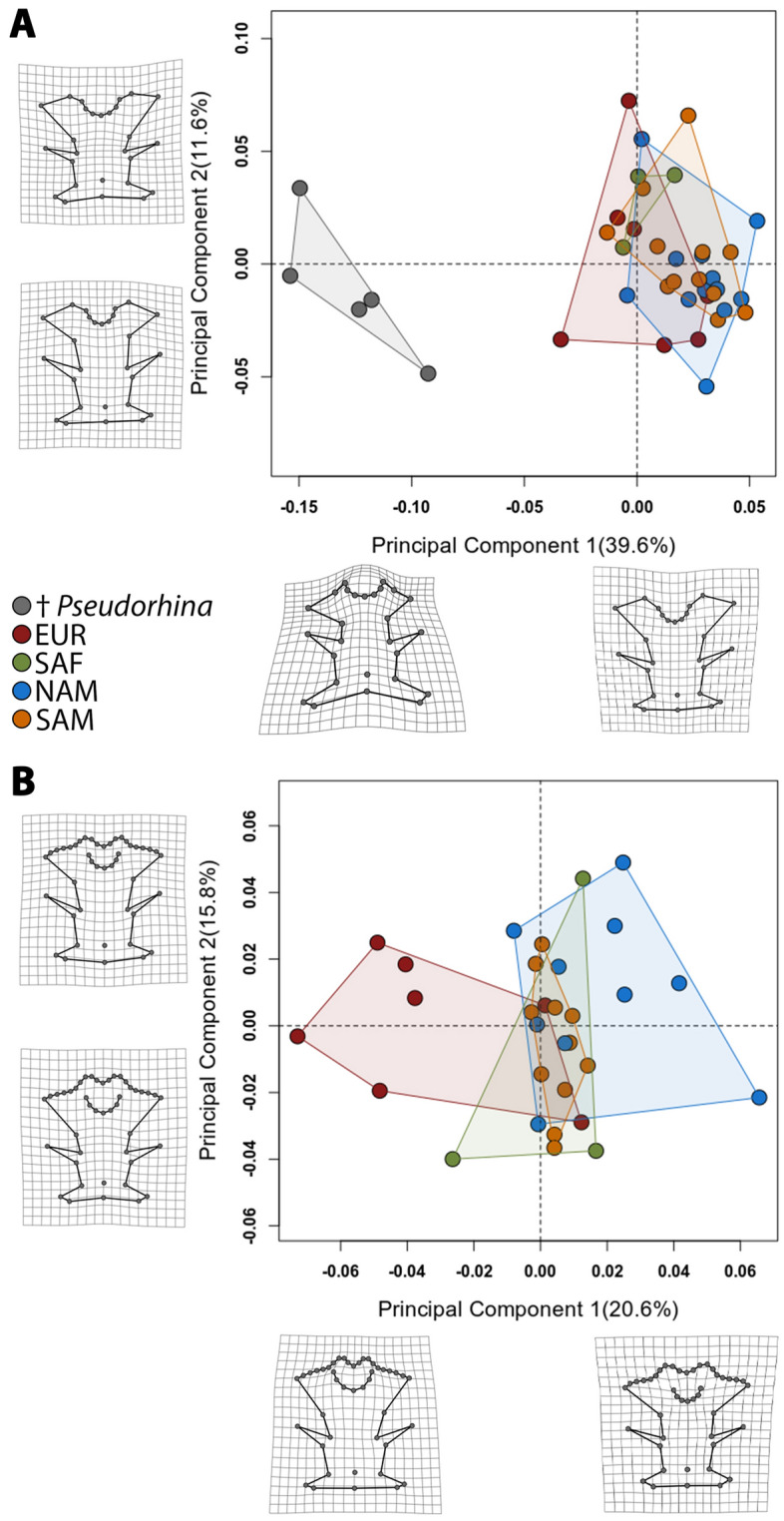
Morphospace of the neurocranium for the extant clades and fossil angel sharks (**A**). Morphospace of the neurocranium of only the extant clades of angel sharks (**B**). Deformation grids indicate the minimum and maximum value of the principal component 1 and 2.

To understand the shape diversity in the neurocranium of Squatiniformes through time and in a spatial context, we studied patterns of disparity and morphological evolutionary rate for the group. The goals of this study are to (1) explore the disparity of both †*Pseudorhina* and *Squatina*, and among the clades within extant *Squatina* (Fig. 1A), (2) observe whether clade arrangement is reflected in morphospace occupation, (3) whether the neurocranial shape bears a phylogenetic signal for the shape variation, and (4) whether noticeable morphological evolutionary rate changes occurred or did the neurocranial shape remain constant through time. We tested whether the neurocranium could be divided into multiple modules to investigate which of these might contribute to the diversity observed in extant clades, and finally we examined whether the level of integration among modules restricts morphological evolution. Taken together, this will provide a better understanding of diversification patterns within this group of unusual sharks.

## Results

### Neurocranium shape among extant and fossil angel sharks

For the geometric morphometrics analyses, we divided the sets into one including †*Pseudorhina acanthoderma* and *Squatina* spp. and another one for only extant *Squatina.* Within *Squatina* the species were divided in European (EUR), African (SAF), North American (NAM) and South American (SAM) clades. The first analysis with the complete data set shows a clear separation of the genera (Fig. 2A). The variation explained by the first four principal components is about 65.5%, with the first and second explaining 51.2%. The morphospace described by the first two principal components indicates that the negative scores of the PC1 define the position of the nasal capsules and preorbital processes, which are directed posteriorly and more laterally. In addition, the anterior fontanelle is wider and appears to be located more anteriorly. Other features include the narrowing of the supraorbital flange, shorter postorbital processes and a wider occipital region, as displayed by the deformation grid (Fig. 2A).

With respect to the positive scores of PC1, the shape changes are defined by the anterolateral projection of the preorbital processes, which clusters the specimens belonging to *Squatina*. The position of the nasal capsule junction with the rostral processes is also located more anteriorly than the anterior fontanelle, which is also narrower compared to specimens of †*Pseudorhina*. Overall, the shape is broader anteriorly comprising the nasal capsules and the anterior fontanelle (Fig. 2A). The supraorbital flange is also broader and the postorbitals are directed more laterally. Finally, the occipital region shows a narrower configuration. Overall these traits defined by the PC1 scores cluster the extant angel sharks. Principal component 2 shows changes related to the position of the supraorbital flange, with no clear separation for any group analysed. On the negative scores, the landmark position of the supraorbital flanges is located more anteriorly, along with a narrow anterior fontanelle and more laterally projected preorbitals. The postorbitals are situated more anteriorly. In the positive values, the anterior fontanelle is broader, and the supraorbital flange is located more posteriorly. Despite the fact that the third and fourth principal components also explain above 5% of the variation, the morphospaces defined by them show redundancy, which does not separate clearly the rest of the groups (Supplementary Fig. S1).

Based on this analysis, it was not possible to find axes of variation to separate the extant angel sharks by clades or species. Thus, we performed another analysis with only the extant *Squatina* spp. and including more semilandmarks for the anterior margin of the rostral processes, since this feature is not preserved in the fossil specimens. Overall, the PCA revealed that the first six PCs explain 71.8% of the total variation. Although the first two PCs only account for 36.4% of the variation, these are the ones that more clearly separate the clades in the morphospace, with a marked overlapping of the SAM and SAF clades in the middle (Fig. 2B). The left side of the morphospace captures a broader anterior fontanelle, shortened rostral processes and the preorbitals directed slightly ventrally. The supraorbital flanges appear to be more separated from the midline, giving a broader appearance to the anterior region of the neurocranium. In addition, the otic region appears broader due to the position of the epiotic crests, which are positioned more laterally compared to the mean shape. On the right side of the morphospace, the rostral processes appear wider and more separated, while the preorbitals are pointing perpendicular relative to the antero-posterior axis of the neurocranium. The anterior fontanelle is narrower and located more posteriorly. The supraorbital flanges are straighter and directed anteriorly, thus the mid-region of the neurocranium appears narrow. The postorbitals are pointing slightly forward and appear more elongated. The otic region also appears more slender and the glossopharyngeal base is directed more anteriorly.

The PC2 shows a very similar pattern for the rostral processes, nasal capsules and preorbitals, as in PC1. The main difference is the anterior fontanelle, which is shorter and narrow in the upper part of the morphospace, and broader and more posteriorly located in the lower morphospace. The supraorbital flanges are positioned more anteriorly in the positive values and the postorbitals are directed forward. A noticeable feature is in the occipital region, where the landmark position shows this neurocranial region is directed posteriorly in the positive scores of the PC. However, this pattern does not seem to be related to a specific feature of a particular clade. As in the case of the previous analysis, the rest of the PCs show a redundancy in features, and therefore cannot separate the groups (Supplementary Fig. S2).

**Figure 3:**
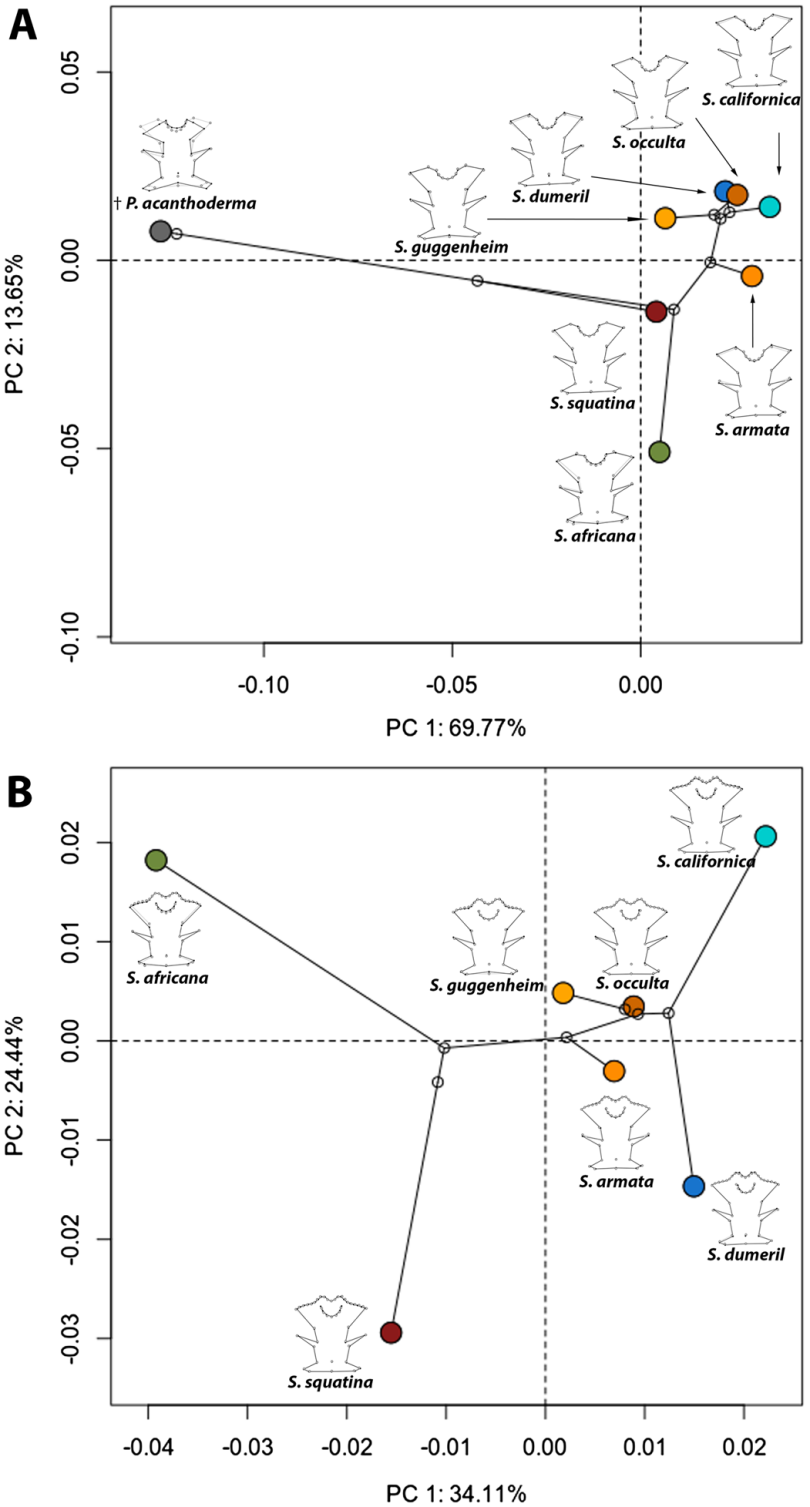
Phylomorphospace for the complete data set of angel sharks (**A**) and extant angel sharks species used in the study (**B**). Mean shape configurations for each species are displayed over the tip of the plotted tree.

**Figure 4:**
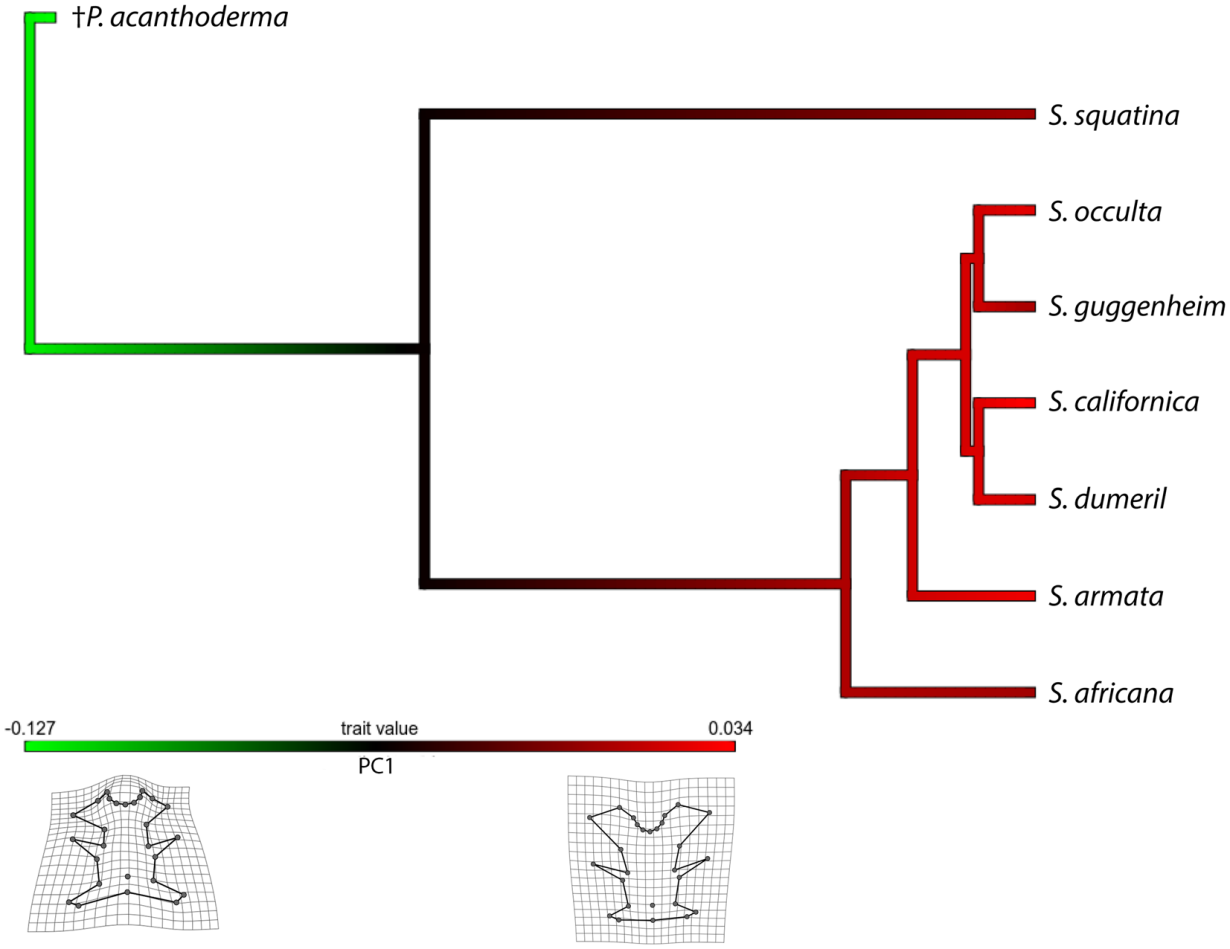
Phylogeny of the Squatiniformes analysed and evolution of the neurocranium. The colours of the branches indicate the shape variation with green towards the shape on the minimum values of the PC1 and in red the maximum values of PC1.

When considering the phylogeny, the phylogenetic signal test is significant for the data set comprising †*Pseudorhina* and only extant *Squatina* (Kmult = 1.4458; p = 0.006 and Kmult = 0.7313; p = 0.0155 respectively). The phylomorphospace for the set with †*Pseudorhina* shows a clear separation of both genera, and both SAM and NAM clades are more closely clustered, while *S. africana* appears to display a different shape of the occipital region compared to the rest of the species (Fig. 3A). When only extant *Squatina* spp. are analysed in the phylomorphospace, we observe a clear separation of the clades, and despite the significant phylogenetic signal, not all the clades resemble each other, as shown by the location of *S. californica* and *S. dumeril* (Fig. 3B)*.* Among Squatiniformes, the genus *Squatina* is overall characterized by the extreme positive scores of PC1, and this is seen as a single event when plotting the PC1 scores on the phylogeny (Fig. 4). The species exhibiting the most extreme values are also the most derived ones, comprising both NAM and SAM clades. One important aspect to consider, at least for the clade SAM, is that it is paraphyletic, since *S. armata* is not part of the group in the tree but is basal to all the American species.

### Disparity and evolution rate

**Figure 5:**
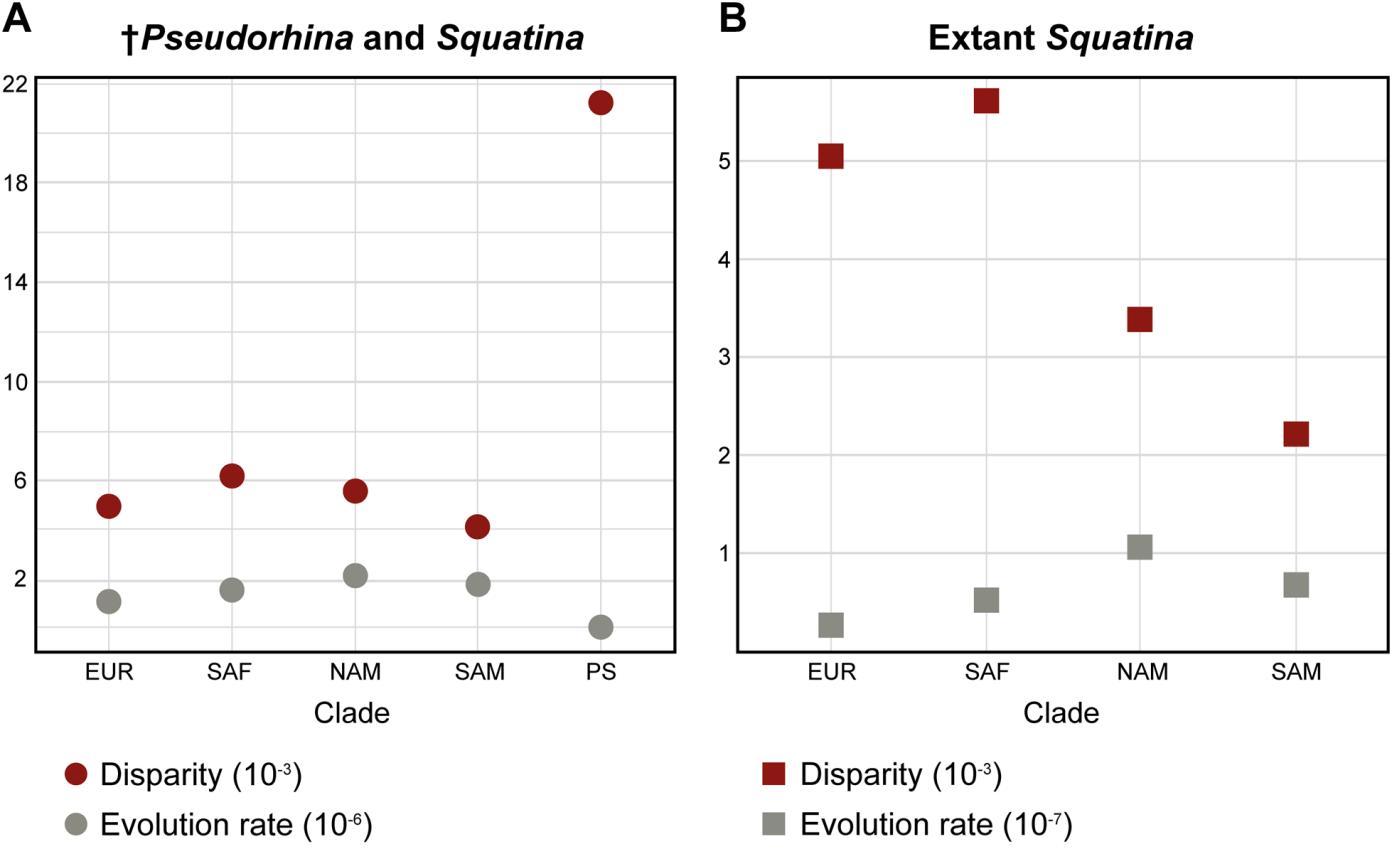
Neurocranial morphological disparity and rate of morphological evolution of the set including the fossil specimens (**A**) and the data set with the living clades of angel sharks (**B**).

Since the PCA for extant *Squatina* displays a geographic pattern for the clades, we investigated how the shape changes are related to this pattern. Following a Procrustes ANOVA, it is evident that the clades are important for defining the shape differences (r^2^ = 0.20366, p = 0.0001). Nevertheless, when considering the phylogeny to define the differences between the clades, the pANOVA indicates no significant differences between the clades (r^2^ = 0.23584, p = 0.996), in spite of a significant phylogenetic signal. When analysing the full data set, the †*Pseudorhina* specimens displayed the largest disparity, followed by SAF and NAM (Fig. 5A). A pairwise test to assess differences shows there are only differences with †*Pseudorhina* compared to the rest. This pattern changes when analysing the extant *Squatina* spp., where SAF and EUR are the species with the highest Procrustes variance (Fig. 5B). The pairwise test also shows that EUR is different from NAM and SAM, but not from SAF. Both SAM and NAM are different from EUR and SAF, but do not differ from each other (Table 1). Likewise, the comparison between species shows that members of related clades are not significantly different from each other, but species from other clades differ as expected. The rates of morphological evolution show that the NAM and SAM clades are the ones with the highest values, although this is not significant (Fig. 5B).

**Table 1:** Pairwise comparisons of the sum of variances for the morphological disparity between the extant clades and the fossil angel shark.

	ENAA	NAM	PS	SAF	SAM
ENAA		0.856	**0.001**	0.775	0.746
NAM	0.000614421		**0.001**	0.898	0.539
PS	0.016309606	0.015695186		**0.001**	**0.001**
SAF	0.001217229	0.000602809	0.01509238		0.641
SAM	0.000870236	0.001484657	0.01717984	0.002087466	

	ENAA	NAM	SAF	SAM	
ENAA		0.065	0.639	**0.001**	
NAM	0.001668977		**0.042**	0.142	
SAF	0.000562415	0.002231392		**0.002**	
SAM	0.002825327	0.00115635	0.003387742		

Upper triangle of values indicates p value and lower triangle the pairwise distance between the variance of each group. Bold values in the upper triangle indicate significant differences at p < 0.05.

### Evolutionary rate of neurocranium modules

**Figure 6:**
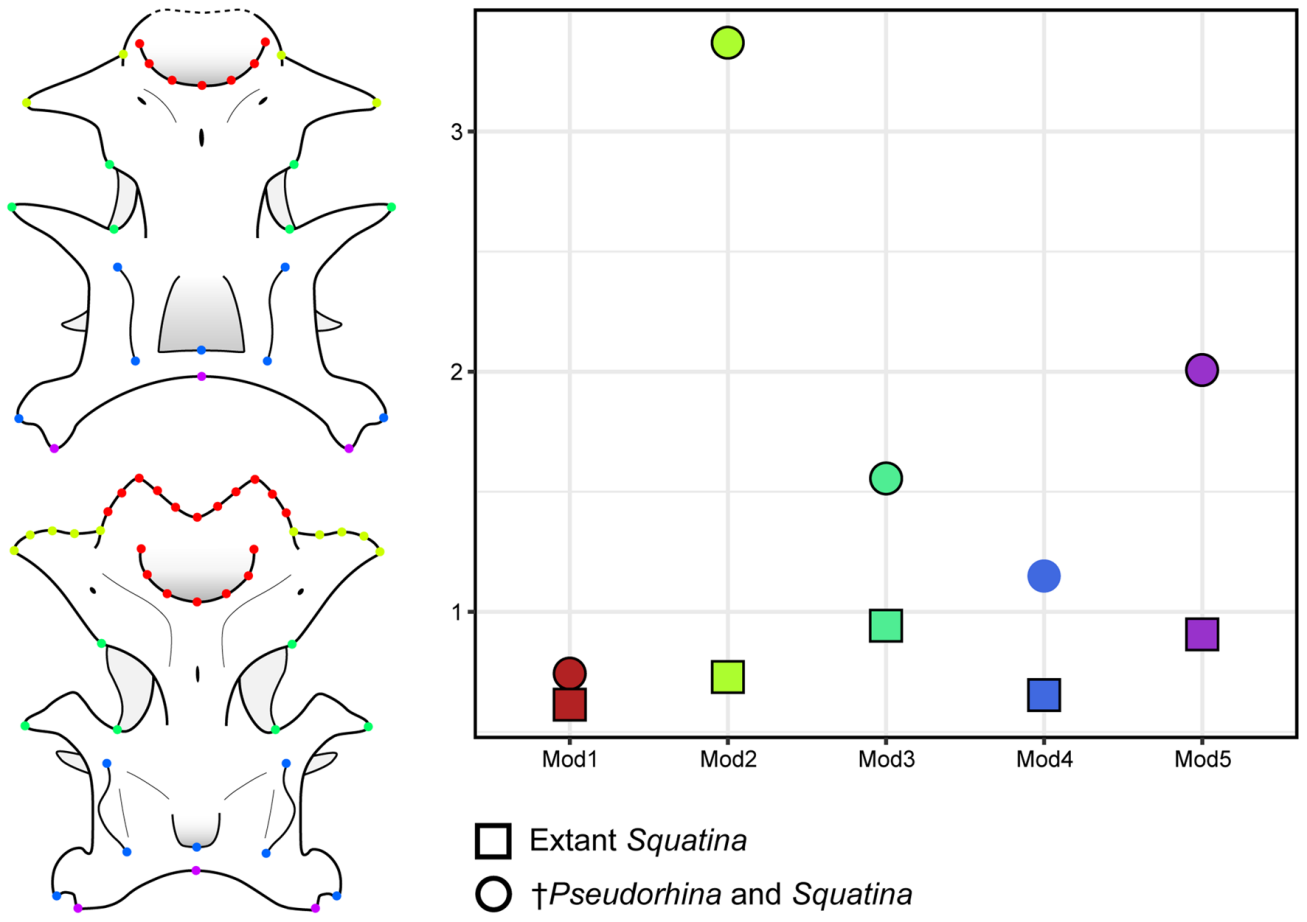
Rate of morphological evolution for selected modules on the neurocranium of the angel sharks, circles indicate the analysis on the complete data set and squares the dataset with only living angel sharks. Modules defined after de Carvalho et al.^27^ Mod1: Rostrum; Mod2: Nasal capsules; Mod3: Orbit; Mod4: Otic capsules; Mod5: Occipital.

Since the values of the phylogenetic signal were significant, we tested for possible patterns of morphological evolution in the different modules of the neurocranium that could indicate certain regions, which are more changeable among the clades. First, we conducted a modularity test on the partitions of landmarks in the neurocranium, followed by integration test in both data sets. The covariance ratio coefficient (CR) for the fossil and extant data set is CR = 0.8496 (p = 0.001), which suggest independence between the modules. Similar results were found in the data set of extant species (CR = 0.6699; p = 0.001). The relatively higher CR value in the complete data set suggest that there might be integration in the modules. Indeed, the integration test for the complete and extant only sets indicates that there is a relatively stronger integration in the complete data set, the partial least squares correlation (r-PLS) is 0.761 (p = 0.001) compared to the extant data set (r-PLS = 0.697; p = 0.013). When we compared the evolution rate of each module, the rostral region shows the lowest evolutionary rate, while the preorbital in the data set with †*Pseudorhina* shows the highest value (Fig. 6). Overall, in the dataset containing only extant *Squatina,* the rate of evolution is lower and, despite the significant phylogenetic signal, the evolutionary rates for the modules are not significant (p = 0.165). This holds also true for the complete data set (p = 1) and can be seen also in the global integration. When analysing the dataset including *†Pseudorhina*, and only extant *Squatina,* the regression slopes (− 0.84823 and − 0.95328, respectively), suggest that the extant species are more integrated than the Late Jurassic squatiniform.

## Discussion

Angel sharks, which show a very consistent dorso-ventrally compressed body plan since their first occurrence in the fossil record during the Late Jurassic^22,23,25^, apparently have experienced three major radiations^46^ finally resulting in their current taxonomic diversity and spatial distribution^17^. However, it is still difficult to clearly differentiate extant angel shark species on the basis of morphological characters, which has led to uncertainty in species designation^27–30,47^. Similarly, many fossil species, which are in most cases based on isolated teeth only, remain dubious^23,25,48^, highlighting their highly conserved morphological nature in their dental traits.

Based on our results, the most noticeable differences in neurocranium shape occurred between †*Pseudorhina* to *Squatina*, with the extant species displaying a very constrained variation, at least in the four clades we analysed. When extant species were analysed separately, a pattern emerged in the levels of disparity between each clade, and geographically close clades displayed non-significant differences. However, this has to be considered with caution, since other clades of angel sharks (from the West Pacific distribution) might show different levels of disparity with respect to the overall group disparity. This can be seen in the rostral projections in *Squatina australis*, which protrude more anteriorly^48^.

Some of the most striking features in the skull of *†Pseudorhina* are the laterally directed preorbitals and postorbitals, also a wider otic region when compared to that of *Squatina* spp. These features clearly separate the two groups in the morphospace. In the morphospace for the extant clades, the shape of the neurocranium becomes more delimited, showing a pattern related to the biogeographic distribution. The EUR clade shows a broader anterior fontanelle and narrower rostral projections. Both the SAM and SAF species display an intermediate phenotype with extensive overlap. In the same way, the differences between both NAM and SAM species are not significant enough to separate both in morphospace. Regarding the disparity, the SAM clade has the lowest levels and also more constrained dispersion in the morphospace, while the NAM clade has a slightly larger disparity. In particular the SAM species have been difficult to distinguish^29^. However, de Carvalho et al.^27^ considered traits like the rostral projections and the anterior fontanelle outline as discrete morphological features suitable for use in species differentiation. In our study, we found that both of these structures display variation enough only to separate the analysed clades, but not at species level. One of the most prominent features of the neurocranium of Squatiniformes are the massive postorbital processes, which in †*Pseudorhina* are slender and in extant *Squatina* spp. display a wide variety of forms^22,23,27,48^. This trait also can be interpreted as a derived feature for modern angel sharks^48^. Despite the wide overlapping shapes in the morphospace, our results revealed that there is a strong phylogenetic signal when analysing the data set including †*Pseudorhina*. This signal, however, is weaker, but still significant if only extant species are considered, which might suggest that for extant species other factors may influence the shape of the neurocranium. Among Squatiniforms, the skull shape can be related to a particular lifestyle displayed by only a few species in all selachians (i.e. elasmobranchs excluding batoids). Angel sharks are bottom dwelling ambush predators showing a particular hiding behaviour^49,50^. Similarly, orectolobiform sharks like wobbegongs (Orectolobidae) are ambush predators displaying flattened bodies, although not as extreme as in angel sharks, and they even have a specialized pigmentation pattern that relate to their ambush behaviour^51–53^. Some of the features in the neurocranium in common between *Orectolobus* and *Squatina* have been described previously^54,55^, such as the occipital condyles and basioccipital fovea. The shared features between these two genera most likely are the result of convergent evolution due to their particular lifestyle.

Based on our results on the phylogenetic signal and ancestral character analysis, we detected that the shape of the skull in angel sharks displays a tendency to similarity in members of a clade. Nevertheless, when we tried to analyse the evolutionary rate of the members of the clades, as well as the evolutionary rate of the different modules of the neurocranium, we were not able to identify any significant differences. Despite the morphological disparity present in the different clades, the lack of significant evolutionary rates indicates that the skull in angel sharks is rather canalised. Some explanations were proposed for the lack of morphological change in spite of speciation, such as phylogenetic niche conservationism^56,57^. At first glance it might appear plausible that angel sharks are not extremely diverse in terms of number of species. However, their fossil record shows that they were distributed from reef-to open marine habitats^22–25,58^. Overall, angel sharks appear to have had a limited capacity for diversification, with only two recognized genera so far.

One of the major forces in shaping morphological disparity is the ecological opportunity, and while angel sharks have been successful to achieve a wide geographical range, they also occupy a restricted zone in the water column. The range of depth in which extant angel sharks occur ranges from 100 to 1390 m^59^. Probably fossil angel sharks were benthopelagic sharks predominantly bound to marginal marine waters similar to the occurrence of extant members. It is possible that other sources of competition could have constrained the opportunities for Squatiniformes to diversify, despite the fact that *Squatina* species are not highly specialised with a restricted dietary niche. For instance, †*Protospinax*, which is a speciose, but still problematic shark ranging from the Early Jurassic to the Late Cretaceous, also displays a highly dorso-ventrally flattened body plan with enlarged pectoral and pelvic fins similar to angel sharks^12,23,58^. This body plan, together with presence of teeth that correspond to the crushing-type, suggests that †*Protospinax* was a benthopelagic shark feeding on various thin- to hard-shelled invertebrates. Interestingly, both †*Protospinax* and †*Pseudorhina* commonly co-occur in the same deposits, which, together with their very similar body plans, might suggests that both genera had similar lifestyles, occupying closely related ecological niches.

Batomorphs, whose fossil record dates back to the Early Jurassic^60,61^ apparently witnessed a major adaptive radiation during the Early Cretaceous^62^. Unlike angel sharks, with which they share a dorso-ventrally compressed body plan, batomorphs have been able to colonize a broad variety of marine habitats, ranging from open marine, offshore to even freshwater environments^18^, due to innovations in their jaw suspension^63,64^ and even swimming mechanisms^65,66^.

It has been shown that higher morphological disparity at a clade origin is usually followed by a decrease in disparity through time^43,45,67^, which might be related to stabilizing selection^68^ and quite often highly specialized behaviours (prey capture for instance) can lead to limited disparities^8,69^. Our results suggest patterns of higher disparity in earlier members among angel sharks. The capacity of angel sharks to diversify in shape could also be a constraint. This means that despite available resources, ecological opportunity, and colonization of novel environments, these are not noticeably reflected in their morphology. The clear biogeographic pattern for extant *Squatina* species, along with their diversification in recent times, can lead to a lack of phenotypic disparity through geographical isolation due to the similar environments that were colonized^35^. The data presented here indicate that the evolutionary rates of the neurocranium in squantiniforms are not significant, which is also reflected in the high integration pattern, although the neurocranium consists of distinct modules. This is more evident when considering the global integration results, where the data set for the extant *Squatina* spp. showed a higher level of integration than the data including †*Pseudorhina*. A higher integration can lead to a constrained ability for novel morphologies^70^. Some of the limitations can come from developmental constraints, in which processes during embryogenesis can become limited in the possible variation^38,71,72^. Only one study has documented the embryonic development in one angel shark species (*Squatina californica*). From the description it is possible to conclude that some of the traits that become apparent later in development are the protrusion of the rostrum and other changes in shape of the head, while the expansion of the pectoral fins and the flattening of the body develop earlier^73^. The body plan in angel sharks is unique among selachians, and this is only shared with other less diverse groups such as the sawsharks and wobbegongs^26^. Other groups of sharks like hammerheads and wobbegongs also display unusual neurocranial shapes, which are related to the sensory system distribution, which could impose a constraint^9,74^. Indeed, interactions of the skull and associated sensory tissues during development suggest a common pattern for such relationships^75,76^.

## Conclusions

Our results indicate that there is a constrained and limited morphological disparity in angel sharks. Certainly, the phenotypic disparity and morphological evolutionary rate might not be always correlated^41,77–79^. The Squatiniformes, with its single extant genus *Squatina*, possess a set of morphological and behavioural traits for their specialized bottom-dwelling ambush predatory lifestyle. The reduced diversity of angel sharks we observe today might be the result of a combination of the factors described above, which ultimately might have led to a restricted niche even since the time they diverged from †*Pseudorhina*. The major driver for the morphological disparity of extant squatiniforms is seemingly only geographic isolation. This is coupled with a higher integration in the extant species, which might limit the evolution between the neurocranium modules.

## Material and methods

### Material

The neurocrania of members of the European (EUR) (n = 7), African (SAF) (n = 3), North American (NAM) (n = 12) and South American (SAM) (n = 12) clades within Squatiniformes^17^ were investigated from published micro-CT scans, published illustrations, museum specimen X-rays, and fossil specimens directly examined by us (Supplementary Table S1). Specimens of †*Pseudorhina* (PS) (n = 5) all belong to †*Pseudorhina acanthoderma*, which is the only species known to be preserved in dorsal view, providing detailed information about the shape of the neurocranium. Only these fossil specimens were used for the analysis, because they produced more reliable placements of landmarks for the downstream analysis. Anatomical nomenclature follows de Carvalho et al.^27^.

### Geometric morphometrics

**Figure 7:**
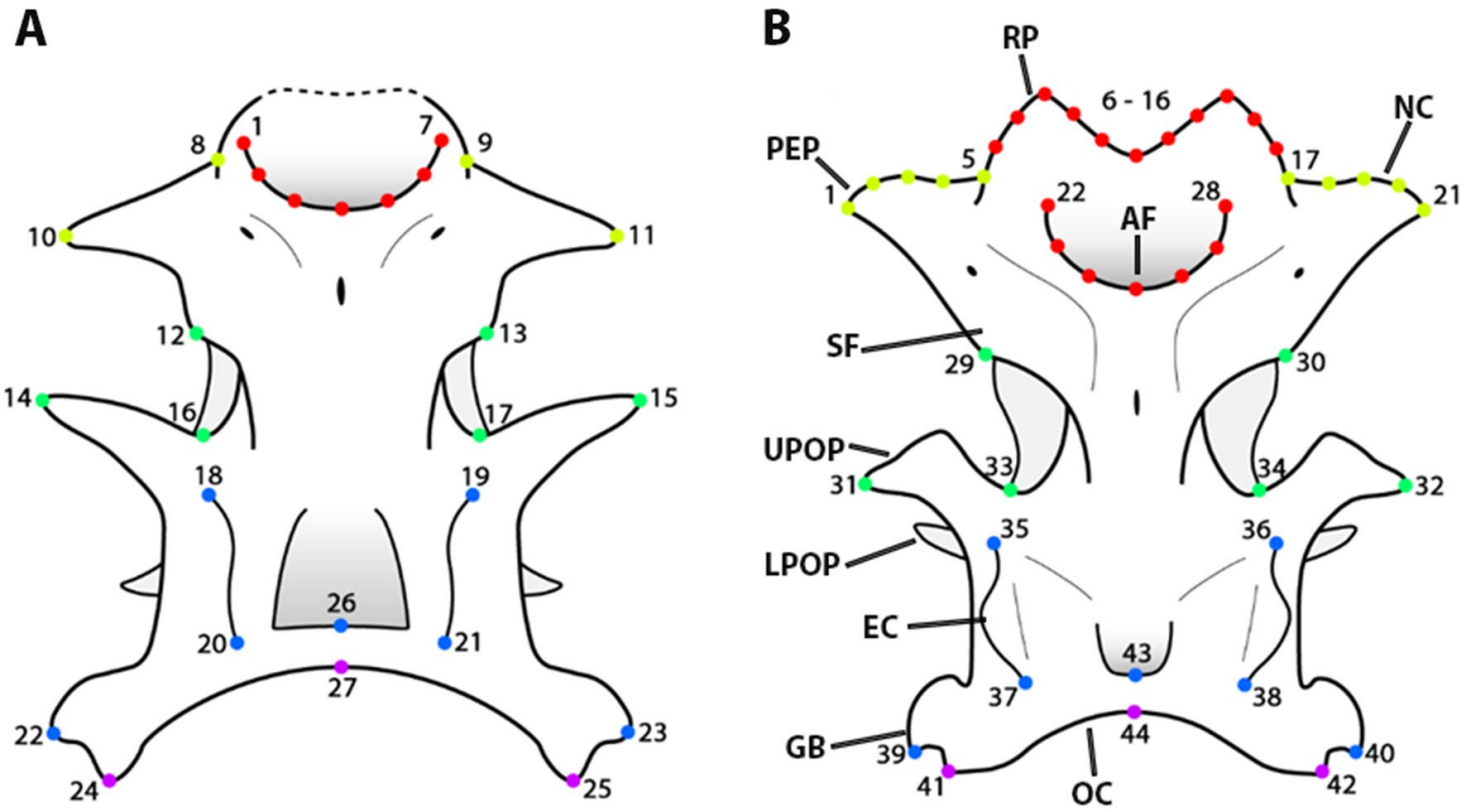
Dorsal view drawings of the neurocranium morphology of †*Pseudorhina* and *Squatina*. Landmark configuration on the neurocranium for the data set including †*Pseudorhina acanthoderma* (**A**). Landmark configuration on the neurocranium for the data set including only the extant species of *Squatina* (**B**). Definitions of the landmarks are detailed in Supplementary Table S1. *AF* anterior fontanelle, *EC* epiotic crest, *GB* glossopharyngeal base, *LPOP* lower postorbital process, *NC* nasal capsules, *OC* occipital, *PEP* preorbital process, *SF* supraorbital flange, *RP* rostral projections, *UPOP* upper postorbital process. Colours indicate the partitions of the modules.

Twenty landmarks and seven semilandmarks for the dataset comprising representatives of the EUR, SAF, NAM, SAM clades, as well as †*Pseudorhina*, were designated to describe the overall shape of the neurocranium (Fig. 7A) (see Supplementary Table S2). In incompletely preserved specimens of †*Pseudorhina*, the landmark coordinates from missing structures were estimated using the function *estimate.missing* in geomorph R package (version 3.1.3^80^). To examine a possible effect for the deformation due to taphonomic bias, we evaluated the pattern of asymmetry with a Procrustes ANOVA, the variation between individuals, variation between the sides, and the interaction term of individuals*sides which reflects fluctuating asymmetry, from this analysis the individual variation in our sample accounts for a larger proportion than the asymmetry. We then evaluated if the asymmetric component would have an effect on the clades, and compared it to the model without asymmetry, from this test we observed that the asymmetric component has a weak effect on the clades as most of the individuals (including the fossils) display asymmetry (Supplementary Table S3). Since the anterior rostral processes are not preserved in any of the available fossil specimens, another landmark configuration including 21 semilandmarks was established for extant *Squatina* spp. to include the most anterior margin of the rostral processes and nasal capsules region (Fig. 7B). The coordinates were captured with the software tpsDig2 (version 2.31^81^). Once the missing landmarks were estimated, they were subjected to a generalized Procrustes analysis to reduce differences in size, orientation, and position^82^, the semilandmarks were slid to reduce the bending energy^83^. The mean shape for each clade and species was also estimated. A Principal component Analysis (PCA) was performed to observe the shape variation in the morphospace for each clade. The mean values of each species were plotted on a phylogeny with the *plotGMPhyloMorphospac*e function to visualize the relationship of shape to the phylogeny of the species analysed. Size can account for the individual shape variation due to the effect of ontogeny, but we found that the log transformed centroid size effect was r^2^ = 0.04446 and not significant (P = 0.1031). Consequently, we did not consider size for further analysis. To evaluate if the neurocranial shape differs between clades, we used a permutational ANOVA followed by a pairwise test of the Procrustes distances variance between clades and species. A phylogenetically informed ANOVA (pANOVA) was performed to test the effect of the clade on the neurocranium shape of extant *Squatina* spp. The sum of variances was estimated with the function *morphol.disparity* and it was used to assess the level of disparity for each clade^84^.

### Phylogenetic signal

We used the molecular phylogeny of Stelbrink et al.^17^ and pruned the tree with the package phytools^85^ to include only the species we analysed to examine how shape variation is reflected on the phylogeny. We estimated the position of †*Pseudorhina* with the function *locate.yeti* from the aggregated mean values of the species, after a PCA with the landmark coordinates. The resulting tree was re-rooted to †*Pseudorhina* in FigTree (version 1.4.4^86^). Finally, we calibrated the tree with the function *chronos* using the package ape (version 5.3^87^). Another tree was used with only extant species of *Squatina* for further analyses. The multivariate K-value (Kmult)^88^ was used to estimate how closely related species within recent clades of *Squatina* resemble each other. We considered a K-value > 1 as an indicator of strong phylogenetic signal and considered significant at α = 0.01.

### Rate of morphological evolution

The landmark configurations were subject to subdivisions reflecting possible hypotheses of modularity, i.e. low covariation between subsets of landmarks^89^. The module partitions are as follows: rostrum, nasal capsules and optic, otic and occipital regions. All the configurations were estimated from the partitions displayed in Fig. 7, and the partitions subject to modularity and integration tests in geomorph with 999 iterations. Additional hypotheses of modules arrangements were tested to reflect possible associations with, for instance embryonic origin of the cartilage, however these resulted as significant as the final one presented here (Supplementary Table S4). To compare the rate of morphological evolution in the clades and also in the module partitions under a Brownian motion model, the functions *compare.mult.evol.rates* and *compare.evol.rates* were used. Finally, we estimated the global integration for the two landmark data sets to infer possible patterns of integration in the neurocranium. This procedure estimates the regression slope of the log of the variance of partial warps against the log of the bending energies^90^. The slopes are used to estimate the integration, with values greater than − 1 suggesting global integration.

## Supplementary information

Supplementary Information.

## Data Availability

All specimens are deposited in collections publicly accessible, indicated in Supplementary Table S1

## References

[1] Coates MI (2003). The evolution of paired fins. Theory Biosci..

[2] Maisey JG (2012). What is an ‘elasmobranch’? The impact of palaeontology in understanding elasmobranch phylogeny and evolution. J. Fish Biol..

[3] Cole NJ, Currie PD (2007). Insights from sharks: evolutionary and developmental models of fin development. Dev. Dyn..

[4] Wilga CA, Ferry LA, Shadwick RE, Farrell AP, Brauner CJ (2015). Functional anatomy and biomechanics of feeding in elasmobranchs. Fish Physiology.

[5] Coates MI (2018). An early chondrichthyan and the evolutionary assembly of a shark body plan. Proc. R. Soc. B..

[6] Sternes PC, Shimada K (2020). Body forms in sharks (Chondrichthyes: Elasmobranchii) and their functional, ecological, and evolutionary implications. Zoology.

[7] Ebert DA, Wilms HA (2013). *Pristiophorus lanae* sp. nov., a new sawshark species from the western North Pacific, with comments on the genus Pristiophorus Müller & Henle, 1837 (Chondrichthyes: Pristiophoridae). Zootaxa.

[8] Gallagher AJ, Hammerschlag N, Shiffman DS, Giery ST (2014). Evolved for extinction: the cost and conservation implications of specialization in hammerhead sharks. Bioscience.

[9] Mara KR, Motta PJ, Martin AP, Hueter RE (2015). Constructional morphology within the head of hammerhead sharks (Sphyrnidae). J. Morphol..

[10] Welten M, Smith MM, Underwood CJ, Johanson Z (2015). Evolutionary origins and development of saw-teeth on the sawfish and sawshark rostrum (Elasmobranchii; Chondrichthyes). R. Soc. Open Sci..

[11] Shirai S (1992). Phylogenetic relationships of the angel sharks, with comments on elasmobranch phylogeny (Chondrichthyes, Squatinidae). Copeia.

[12] de Carvalho MR, Maisey JG, Arratia G, Viohl G (2008). Phylogenetic relationships of the Late Jurassic shark *Protospinax* Woodward 1919 (Chondrichthyes: Elasmobranchii). Mesozoic Fishes 1: Systematics and Paleoecology.

[13] Douady CJ, Dosay M, Shivji MS, Stanhope MJ (2003). Molecular phylogenetic evidence refuting the hypothesis of Batoidea (rays and skates) as derived sharks. Mol. Phylogenet. Evol..

[14] Vélez-Zuazo X, Agnarsson I (2011). Shark tales: a molecular species-level phylogeny of sharks (Selachimorpha, Chondrichthyes). Mol. Phylogenet. Evol..

[15] Naylor, G. J. P. et al. in *The Biology of Sharks and Their Relatives* (eds Carrier, J. C., Musick, J. A. & Heithaus, M. R.) 31–56 (CRC Press, Boca Raton, FL, 2012).

[16] Amaral CR, Pereira F, Silva DA, Amorim A, de Carvalho EF (2018). The mitogenomic phylogeny of the Elasmobranchii (chondrichthyes). Mitochondrial. DNA A..

[17] Stelbrink B, von Rintelen T, Cliff G, Kriwet J (2010). Molecular systematics and global phylogeography of angel sharks (genus *Squatina*). Mol. Phylogenet. Evol..

[18] Compagno L, Dando M, Fowler S (2005). Sharks of the World.

[19] Castro-Aguirre JL, Espinosa Pérez H, Huidobro Campos L (2006). Dos nuevas especies del género *Squatina* (Chondrichthyes: Squatinidae) del Golfo de México. Rev. Biol. Trop..

[20] Last PR, White WT (2008). Three new angel sharks (Chondrichthyes: Squatinidae) from the Indo-Australian region. Zootaxa.

[21] Acero PA, Tavera JJ, Anguila R, Hernández L (2016). A new southern Caribbean species of angel shark (Chondrichthyes, Squaliformes, Squatinidae), including phylogeny and tempo of diversification of American species. Copeia.

[22] de Carvalho MD, Kriwet J, Thies D, Arratia G, Schultze HP, Wilson VH (2008). A systematic and anatomical revision of Late Jurassic angelsharks (Chondrichthyes: Squatinidae). Mesozoic Fishes 4: Homology and Phylogeny.

[23] Klug S, Kriwet J (2013). Node age estimations and the origin of angel sharks, Squatiniformes (Neoselachii, Squalomorphii). J. Syst. Palaeontol..

[24] Guinot G, Underwood CJ, Cappetta H, Ward DJ (2012). Squatiniformes (Chondrichthyes, Neoselachii) from the Late Cretaceous of southern England and northern France with redescription of the holotype of *Squatina cranei* Woodward, 1888. Palaeontology.

[25] Underwood CJ (2002). Sharks, rays and a chimaeroid from the Kimmeridgian (Late Jurassic) of Ringstead, southern England. Palaeontology.

[26] Ducatez S (2019). Which sharks attract research? Analyses of the distribution of research effort in sharks reveal significant non-random knowledge biases. Rev. Fish Biol. Fish..

[27] de Carvalho MR, Faro C, Gomes UL (2012). Comparative neurocranial morphology of angelsharks from the south-western Atlantic Ocean (Chondrichthyes, Elasmobranchii, Squatinidae): implications for taxonomy and phylogeny. Acta Zool..

[28] Walsh JH, Ebert DA (2007). A review of the systematics of western North Pacific angel sharks, genus *Squatina*, with redescriptions of *Squatina formosa*, *S. japonica*, and *S. nebulosa* (Chondrichthyes: Squatiniformes, Squatinidae). Zootaxa.

[29] Vaz DF, de Carvalho MR (2013). Morphological and taxonomic revision of species of *Squatina* from the Southwestern Atlantic Ocean (Chondrichthyes: Squatiniformes: Squatinidae). Zootaxa.

[30] Vaz DF, de Carvalho MR (2018). New Species of *Squatina* (Squatiniformes: Squatinidae) from Brazil, with Comments on the Taxonomy of Angel Sharks from the Central and Northwestern Atlantic. Copeia.

[31] Harmon LJ, Melville J, Larson A, Losos JB (2008). The role of geography and ecological opportunity in the diversification of day geckos (*Phelsuma*). Syst. Biol..

[32] Rundell RJ, Price TD (2009). Adaptive radiation, nonadaptive radiation, ecological speciation and nonecological speciation. Trends Ecol. Evol..

[33] West-Eberhard MJ (2003). Developmental Plasticity and Evolution.

[34] Funk DJ, Nosil P, Etges WJ (2006). Ecological divergence is consistently positively associated with reproductive isolation across disparate taxa. Proc. Natl Acad. Sci. USA.

[35] Losos JB, Mahler DL, Bell MA, Futuyma DJ, Eanes WF, Levinton JS (2010). Adaptive radiation: the interaction of ecological opportunity, adaptation, and speciation. Evolution Since Darwin: The First 150 Years.

[36] Bürger R, Schneider KA, Willensdorfer M (2006). The conditions for speciation through intraspecific competition. Evolution.

[37] Ghalambor CK, McKay JK, Carroll SP, Reznick DN (2007). Adaptive versus non-adaptive phenotypic plasticity and the potential for contemporary adaptation in new environments. Funct. Ecol..

[38] Willmore KE, Young NM, Richtsmeier JT (2007). Phenotypic variability: its components, measurement and underlying developmental processes. Evol. Biol..

[39] Lovette IJ, Bermingham E, Ricklefs RE (2002). Clade-specific morphological diversification and adaptive radiation in Hawaiian songbirds. Proc. R. Soc. Lond. B..

[40] Barluenga M, Stölting KN, Salzburger W, Muschick M, Meyer A (2006). Sympatric speciation in Nicaraguan crater lake cichlid fish. Nature.

[41] Adams DC, Berns CM, Kozak KH, Wiens JJ (2009). Are rates of species diversification correlated with rates of morphological evolution?. Proc. R. Soc. B. Biol. Sci..

[42] Foote M (1997). The evolution of morphological diversity. Annu. Rev. Ecol. Syst..

[43] Erwin DH (2007). Disparity: morphological pattern and developmental context. Palaeontology.

[44] Friedman M (2010). Explosive morphological diversification of spiny-finned teleost fishes in the aftermath of the end-Cretaceous extinction. Proc. R. Soc. Lond. B..

[45] Hughes M, Gerber S, Wills MA (2013). Clades reach highest morphological disparity early in their evolution. Proc. Natl Acad. Sci. USA.

[46] Kriwet J, Benton MJ (2004). Neoselachian (chondrichthyes, elasmobranchii) diversity across the cretaceous–tertiary boundary. Palaeogeogr. Palaeoclimatol. Palaeoecol..

[47] Walsh JH, Ebert DA, Compagno LJ (2011). *Squatina caillieti* sp. nov., a new species of angel shark (Chondrichthyes: Squatiniformes: Squatinidae) from the Philippine Islands. Zootaxa.

[48] Mollen FH, van Bakel BW, Jagt JW (2016). A partial braincase and other skeletal remains of Oligocene angel sharks (Chondrichthyes, Squatiniformes) from northwest Belgium, with comments on squatinoid taxonomy. Contrib. Zool..

[49] Fouts WR, Nelson DR (1999). Prey capture by the Pacific angel shark, *Squatina californica*: visually mediated strikes and ambush-site characteristics. Copeia.

[50] Tomita T, Toda M, Murakumo K (2018). Stealth breathing of the angelshark. Zoology.

[51] Carraro R, Gladstone W (2006). Habitat preferences and site fidelity of the ornate wobbegong shark (*Orectolobus ornatus*) on rocky reefs of New South Wales1. Pac. Sci..

[52] Corrigan S, Beheregaray LB (2009). A recent shark radiation: molecular phylogeny, biogeography and speciation of wobbegong sharks (family: Orectolobidae). Mol. Phylogenet. Evol..

[53] Kempster RM, McCarthy ID, Collin SP (2012). Phylogenetic and ecological factors influencing the number and distribution of electroreceptors in elasmobranchs. J. Fish Biol..

[54] Holmgren N (1941). Studies on the head in fishes embryological, morphological, and phylogenetical researches: part II: comparative anatomy of the adult selachian skull, with remarks on the dorsal fins in sharks. Acta Zool..

[55] Claeson KM, Hilger A (2011). Morphology of the anterior vertebral region in elasmobranchs: special focus, Squatiniformes. Foss. Rec..

[56] Wiens JJ, Donoghue MJ (2004). Historical biogeography, ecology and species richness. Trends Ecol Evol..

[57] Losos JB (2008). Phylogenetic niche conservatism, phylogenetic signal and the relationship between phylogenetic relatedness and ecological similarity among species. Ecol. Lett..

[58] Guinot G, Cappetta H, Adnet S (2014). A rare elasmobranch assemblage from the Valanginian (Lower Cretaceous) of southern France. Cretac. Res..

[59] Priede IG, Froese R (2013). Colonization of the deep sea by fishes. J. Fish Biol..

[60] Delsate D, Candoni L (2001). Description de nouveaux morphotypes dentaires de *Batomorphii toarciens* (*Jurassique inférieur*) du Bassin de Paris: Archaeobatidae nov. fam. Bull Soc. Nat. Luxemb..

[61] Stumpf S, Kriwet J (2019). A new Pliensbachian elasmobranch (Vertebrata, Chondrichthyes) assemblage from Europe, and its contribution to the understanding of late Early Jurassic elasmobranch diversity and distributional patterns. PalZ.

[62] Guinot G, Cavin L (2016). ‘Fish’(Actinopterygii and Elasmobranchii) diversification patterns through deep time. Biol. Rev.

[63] Dean MN, Bizzarro JJ, Summers AP (2007). The evolution of cranial design, diet, and feeding mechanisms in batoid fishes. Integr. Comp. Biol..

[64] Wilga CD, Motta PJ, Sanford CP (2007). Evolution and ecology of feeding in elasmobranchs. Integr. Comp. Biol..

[65] Schaefer JT, Summers AP (2005). Batoid wing skeletal structure: novel morphologies, mechanical implications, and phylogenetic patterns. J. Morphol..

[66] Hall KC, Hundt PJ, Swenson JD, Summers AP, Crow KD (2018). The evolution of underwater flight: the redistribution of pectoral fin rays, in manta rays and their relatives (Myliobatidae). J. Morphol..

[67] Frédérich B, Marramà G, Carnevale G, Santini F (2016). Non-reef environments impact the diversification of extant jacks, remoras and allies (Carangoidei, Percomorpha). Proc. R. Soc. B.

[68] Linde-Medina M, Boughner JC, Santana SE, Diogo R (2016). Are more diverse parts of the mammalian skull more labile?. Ecol. Evol..

[69] Holliday JA, Steppan SJ (2004). Evolution of hypercarnivory: the effect of specialization on morphological and taxonomic diversity. Paleobiology.

[70] Klingenberg CP (2008). Morphological integration and developmental modularity. Annu. Rev. Ecol. Evol. Syst..

[71] Arthur W (2001). Developmental drive: an important determinant of the direction of phenotypic evolution. Evol. Dev..

[72] Roux J, Robinson-Rechavi M (2008). Developmental constraints on vertebrate genome evolution. PLoS Genet..

[73] Natanson LJ, Cailliet GM (1986). Reproduction and development of the Pacific angel shark, *Squatina californica*, off Santa Barbara, California. Copeia.

[74] Theiss SM, Collin SP, Hart NS (2011). Morphology and distribution of the ampullary electroreceptors in wobbegong sharks: implications for feeding behaviour. Mar. Biol..

[75] Adameyko I, Fried K (2016). The nervous system orchestrates and integrates craniofacial development: a review. Front. Physiol..

[76] Yang LM, Ornitz DM (2019). Sculpting the skull through neurosensory epithelial–mesenchymal signaling. Dev. Dyn..

[77] Renaud S, Auffray JC, Michaux J (2006). Conserved phenotypic variation patterns, evolution along lines of least resistance, and departure due to selection in fossil rodents. Evolution.

[78] Alhajeri BH, Steppan SJ (2018). Disparity and evolutionary rate do not explain diversity patterns in muroid rodents (Rodentia: Muroidea). Evol. Biol..

[79] Michaud M, Veron G, Peignè S, Blin A, Fabre AC (2018). Are phenotypic disparity and rate of morphological evolution correlated with ecological diversity in Carnivora?. Biol. J. Linnean. Soc..

[80] Adams DC, Otárola-Castillo E (2013). geomorph: an R package for the collection and analysis of geometric morphometric shape data. Methods Ecol. Evol..

[81] Rohlf, F. J. *tps-DIG, Digitize Landmarks and Outlines, Version 231. [Software and Manual].* New York: Department of Ecology and Evolution, State University of New York at Stony Brook (2008).

[82] Rohlf FJ, Slice D (1990). Extensions of the Procrustes method for the optimal superimposition of landmarks. Syst. Biol..

[83] Gunz P, Mitteroecker P (2013). Semilandmarks: a method for quantifying curves and surfaces. HYSTRIX.

[84] Zelditch ML, Swiderski DL, Sheets HD (2012). Geometric Morphometrics for Biologists: A Primer.

[85] Revell LJ (2012). phytools: an R package for phylogenetic comparative biology (and other things). Methods Ecol. Evol..

[86] Rambaut and Drummond, FigTree. https://github.com/rambaut/figtree/releases. 2018

[87] Paradis E (2015). Package ‘ape’. Anal. Phylogeneti. Evol..

[88] Adams DC (2014). A generalized K statistic for estimating phylogenetic signal from shape and other high-dimensional multivariate data. Syst. Biol..

[89] Adams DC (2016). Evaluating modularity in morphometric data: challenges with the RV coefficient and a new test measure. Methods Ecol. Evol..

[90] Bookstein FL (2015). Integration, disintegration, and self-similarity: characterizing the scales of shape variation in landmark data. Evol. Biol..

