# Supplementary material for: Evolutionary trends of the conserved neurocranium shape in angel sharks (Squatiniformes, Elasmobranchii)
Source: Sci Rep. 2020 Jul 28;10:12582. doi: 10.1038/s41598-020-69525-7 (PMC7387474; doi:10.1038/s41598-020-69525-7)
Supplement: Supplementary file 1 — Supplementary Information. [file 41598_2020_69525_MOESM1_ESM.pdf]

Evolutionary trends of the conserved neurocranium shape in angel sharks (Squatiniformes, Elasmobranchii)

Faviel A. López-Romero[1]*, Sebastian Stumpf[1], Cathrin Pfaff[1], Giuseppe Marramà[2], Zerina Johanson[3], Jürgen Kriwet[1]

[1] University of Vienna, Department of Palaeontology, Vienna, Austria.

[2] Università degli Studi di Torino, Dipartimento di Scienze della Terra, Torino, Italy

[3] Natural History Museum, Department of Earth Sciences, Cromwell Road, London, SW75BD UK.

* Correspondence: Faviel A. López-Romero

faviel.alejandro.lopez.romero@univie.ac.at

Supplementary Information

Supplementary Table 1. List of specimens and catalogue number of the individuals used in the present study. Institutional codes: EBR: Elasmobranch Research Belgium, Bonheiden; BMNH: Life Sciences collection, Natural History Museum, London; USNM: Smithsonian National Museum of Natural History, DC; NUPEC: Núcleo de Pesquisas e Estudos em Chondrichthyes, Santos; MZUSP: Museu de Zoologia da Universidade do Estado do Rio de Janeiro, Rio de Janeiro; AMNH: American Museum of Natural History, New York; FSFL: Far Seas Fisheries Research Laboratory, Shimizu; SMNS: Staatliches Museum für Naturkunde Stuttgart, Stuttgart; GPIT: Geologisches und Paläontologisches Institut Tübingen; NMW: Naturhistorisches Museum Wien.

ID	Catalogue number		Clade
†*Pseudorhina acanthoderma*	SMNS369523	Prepared fossil	PS
†*Pseudorhina acanthoderma*	SMNS 8621441	Prepared fossil	PS
†*Pseudorhina acanthoderma*	SMNS8014424	Prepared fossil	PS
†*Pseudorhina acanthoderma*	GPIT8214	Prepared fossil	PS
†*Pseudorhina acanthoderma*	GPIT6842	Prepared fossil	PS
Squatina africana	FSFL-EL205	x-Ray	SAF
Squatina africana	ERB0971	CT-Scan	SAF
Squatina africana	BMNH1906111921	x-Ray	SAF
Squatina armata	USNM RAD110877-001C	x-Ray	SAM
Squatina armata	USNM RAD110877-001A	x-Ray	SAM
Squatina armata	USNM RAD110876-001	x-Ray	SAM
Squatina armata	USNM RAD110875-001	x-Ray	SAM
Squatina armata	USNM RAD110873-001	x-Ray	SAM
Squatina armata	USNM RAD110872-001	x-Ray	SAM
Squatina californica	USNM RAD110882-001	x-Ray	NAM
Squatina californica	USNM RAD110881-001	x-Ray	NAM
Squatina californica	USNM RAD110880-001	x-Ray	NAM
Squatina californica	USNM RAD109873-2	x-Ray	NAM
Squatina californica	USNM RAD109873-1	x-Ray	NAM
Squatina californica	BC02	Skeleton preparation	NAM
Squatina californica	AMNH55686	Skeleton preparation	NAM
Squatina dumeril	USNM410798	x-Ray	NAM
Squatina dumeril	USNM358610	x-Ray	NAM
Squatina dumeril	ERB1026	CT-scan	NAM
Squatina guggenheim	MZUSP110869	Drawing	SAM
Squatina guggenheim	ERB0881	CT-scan	SAM
Squatina guggenheim	AUERJ481	Drawing	SAM
Squatina occulta	NUPEC2192	Drawing	SAM

Squatina occulta	AUERJ792	Drawing	SAM
Squatina squatina	ERB1029	CT-scan	ENAA
Squatina squatina	BMNH91519240	CT-Scan	ENAA
Squatina squatina	USNM188104-2	x-Ray	ENAA
Squatina squatina	USNM188104-1	x-Ray	ENAA
Squatina squatina	USNM157746	x-Ray	ENAA
Squatina squatina	NMW87247sp3	x-Ray	ENAA
Squatina squatina	NMW87247sp2	x-Ray	ENAA

Supplementary Table 2. List of Landmarks for the configurations for the whole data set of individuals (†*Pseudorhina* and *Squatina*) and the data set with only living angel sharks. The order of the landmarks follows the arrangement presented in figure 2.

Landmark configurations		
Pseudorhina and *Squatina* data set		Module
sL1-sL7	Anterior fontanelle curve	M1
L8-L9	Rostral process-Nasal capsule intersection	M2
L10-L11	Tip of the preorbital processes	M2
L12-L13	Supraorbital Flange	M3
L14-L15	Tip of the post orbital process	M3
L16-L17	Base of the post orbital process	M3
L18-L19	Anterior tip of the epiotic crest	M4
L20-L21	Posterior tip of the epiotic crest	M4
L22-L23	Tip of the glossopharyngeal base	M4
L24-L25	Post-otic process	M5
L26	Medial point of parietal fossa	M4
L27	Foramen magnum	M5
Squatina data set		
sL1-sL21	Anterior margin of the Rostrum and Nasal capsules	
	(sL6-sL16) Rostral processes	M1
	(sL1-sL5:sL17-sL21) Nasal capsule-Preorbital processes	M2
sL22-sL28	Anterior fontanelle curve	M1
L29-L30	Supraorbital Flange	M3
L31-L32	Tip of the post orbital process	M3
L33-L34	Base of the post orbital process	M3
L35-L36	Anterior tip of the epiotic crest	M4
L37-L38	Posterior tip of the epiotic crest	M4
L39-L40	Tip of the glossopharyngeal base	M4
L41-L42	Post-otic proces	M5
L43	Medial point of parietal fossa	M4
L44	Foramen magnum	M5

Supplementary Table 3. Procrustes ANOVA on the individual and side variation to estimate fluctuating asymmetry, and shape variation on clades due to the fluctuating asymmetry component ANOVA.

Procrustes ANOVA on object symmetry							
	Df	SS	MS	R^2	F	Z	P - value
ind	38	0.46919	0.0123472	0.85869	6.9629	13.7634	0.001
side	1	0.00983	0.0098295	0.01799	5.5431	3.3807	0.002
ind:side	38	0.06739	0.0017733	0.12332			
Total	77	0.54641					
Procrustes ANOVA with the fluctuating asymmetry component							
clade	4	0.021538	0.0053846	0.15981	1.6168	1.8396	0.035
Residuals	34	0.113232	0.0033304	0.84019			
Total	38	0.134771					

Supplementary Table 4. Covariance Ratio and r-PLS results on different module hypotheses on the neurocranium of Squatiniformes. Comparisons made for the complete data set including fossil specimens, and subset with only extant specimens. Modules arrangements as displayed in supplementary figure 3.

	CR	CR+CI	CR-CI	P	r-PLS	P
Set with fossils						
2Modules	0.8854	0.9821	0.7933	0.006	0.832	0.001
3AModules	0.8939	1.0008	0.7831	0.001	0.814	0.002
3BModules	0.8142	0.9433	0.7342	0.001	0.781	0.008
4AModules	0.7979	0.9219	0.7121	0.001	0.757	0.001
4BModules	0.8239	0.9536	0.7587	0.001	0.735	0.002
5Modules	0.8496	0.9663	0.7605	0.001	0.761	0.001
Set with only extant						
2Modules	0.7246	0.8817	0.6856	0.001	0.844	0.001
3AModules	0.7134	0.8622	0.7036	0.001	0.806	0.006
3BModules	0.6462	0.8216	0.6507	0.001	0.717	0.026
4AModules	0.5991	0.7729	0.6133	0.001	0.689	0.011
4BModules	0.7209	0.8789	0.7043	0.001	0.717	0.005
5Modules	0.6699	0.8321	0.6679	0.001	0.697	0.002

CR: Covariance ratio, CI: Confidence interval, P: P-value, r-PLS: correlation coefficient of the two-block partial least squares.

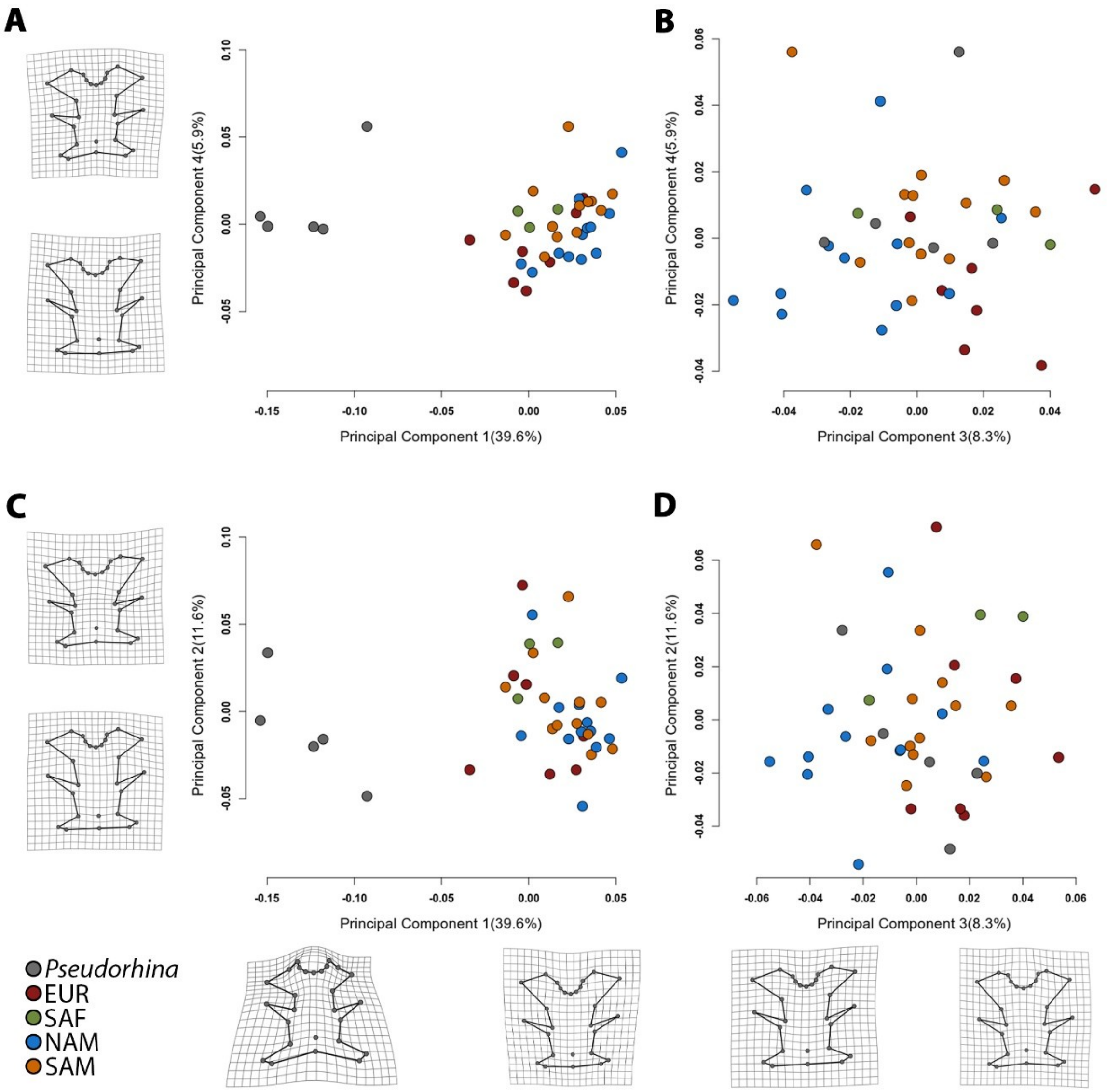

Supplementary Figure S1. Comparison of the first four principal components for the data set with †*Pseudorhina*. (A) PC1 and PC4, (B) PC3 and PC4, (C) PC1 and PC2, (D) PC2 and PC3.

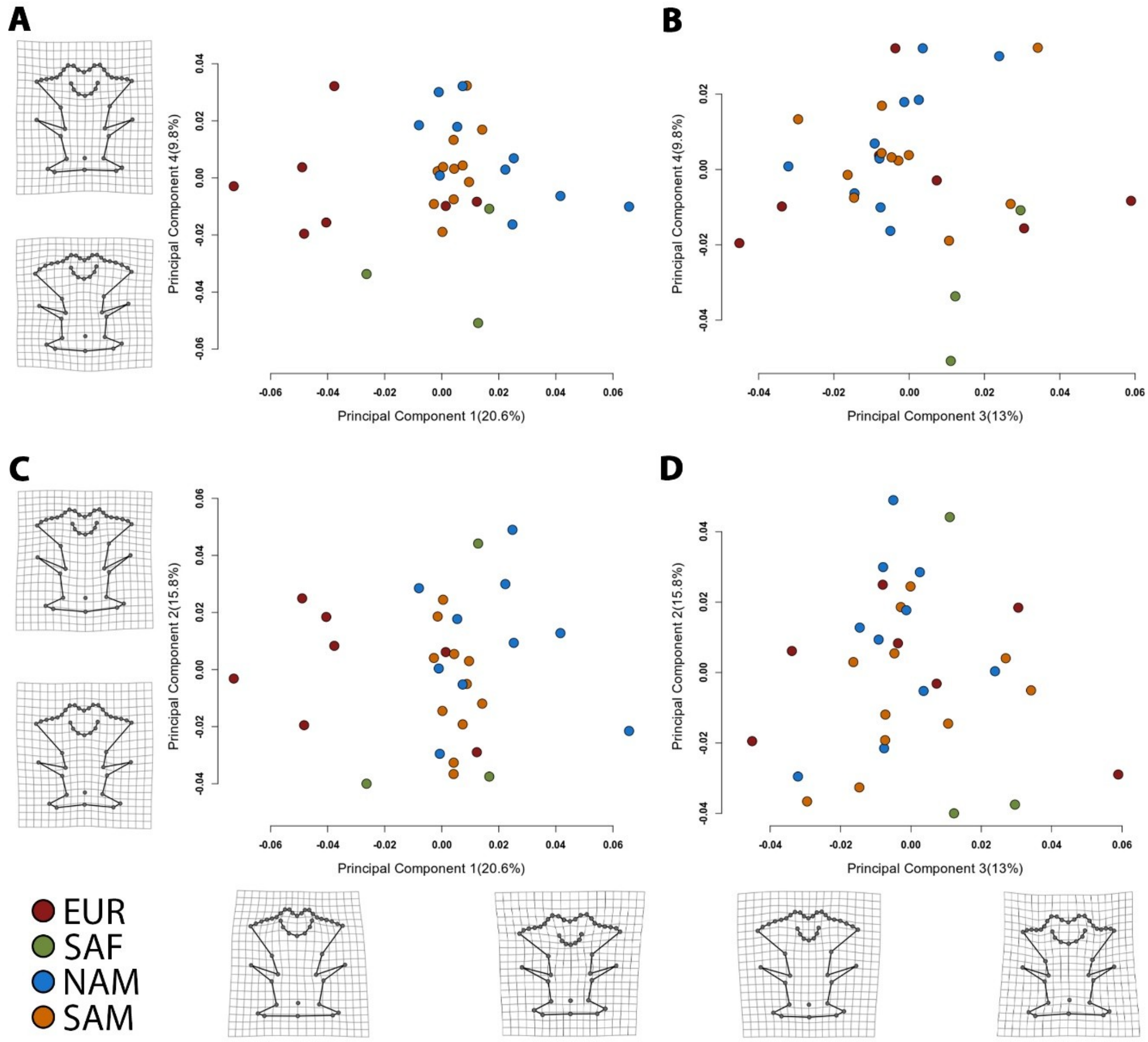

Supplementary figure 2. Comparison of the first four principal components of the extant angel sharks by clade. (A) PC1 and PC4, (B) PC3 and PC4, (C) PC1 and PC2, (D) PC2 and PC3.

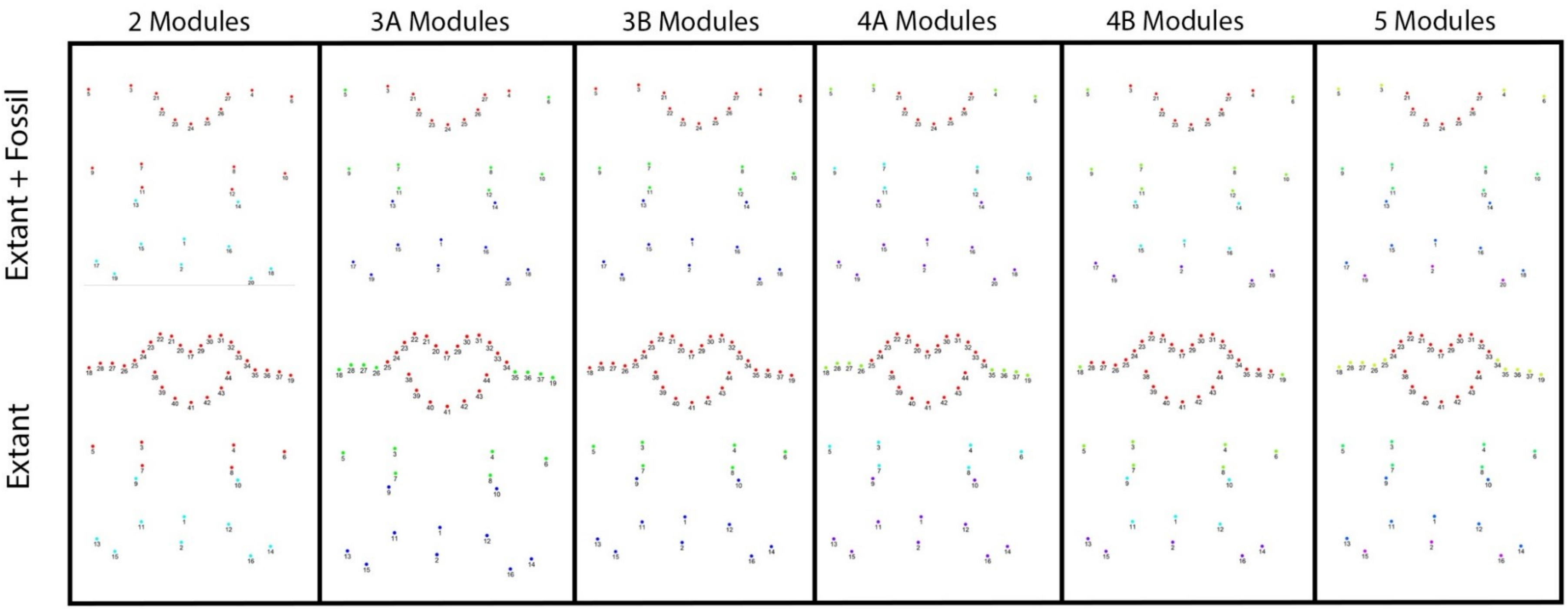

Supplementary figure 3. Alternative modularity hypotheses tested for the complete data set including fossil specimens, and for the extant specimens only. Hypothesis with 5 modules is the used in the main text. Colours indicate the landmarks assigned to different modules.
